# Chair of Chemistry in the University of Edinburgh

**Published:** 1858-09

**Authors:** 


CHAIR OF CHEMISTRY IN THE UNIVERSITY OF EDINBURGH.—The candidates for this highly honorable and lucrative position, rendered vacant by the death of Professor William Gregory, were, at first, Dr. George Wilson, Regius Professor of Technology in the same institution, and Dr. Thomas Anderson, Professor of Chemistry in the Glasgow University. Dr. Wilson, however, withdrew, leaving Dr. Lyon Playfair and Dr. Anderson the prominent candidates. On the 28th of June, the Town Council held an election, resulting in the choice of Dr. Playfair by a majority of sixteen votes. This appointment will doubtless be a source of gratification to the Alumni and students of the institution. Dr. Playfair is a fluent lecturer and a good teacher, being well versed in all the branches pertaining to chemical science.

